# Clinical response to Auron Misheil Therapy in a man with advanced multifocal hepatocellular carcinoma: A case report

**DOI:** 10.1186/1752-1947-5-478

**Published:** 2011-09-24

**Authors:** Jürgen S Scheele, Jan Harder, Zoran Stankovic, Daniel Räpple, Annette Dorn, Hans C Spangenberg, Hubert E Blum

**Affiliations:** 1 Department of Hematology and Oncology, University Medical Center Freiburg, Hugstetter Strasse 55, 79106 Freiburg, Germany; 2 II Medical Clinic, Hegau-Bodensee-Klinikum Singen, Vierchowstrasse 10, 78224 Singen, Germany; 3 Department of Radiology, University Medical Center Freiburg, Hugstetter Strasse 55, 79106 Freiburg, Germany; 4 Department of Medicine II, University Medical Center Freiburg, Hugstetter Strasse 55, 79106 Freiburg, Germany

## Abstract

**Introduction:**

Auron Misheil Therapy was developed based on similarities between carcinogenesis and inflammation. Auron Misheil Therapy is a combination of natural and synthetic compounds, including anti-inflammatory drugs and insulin, expected to exhibit synergistic effects.

**Case presentation:**

Here, we report the case of a 78-year-old Caucasian male patient who presented with multifocal hepatocellular carcinoma and chronic hepatitis C virus infection. Over a four-year period our patient was treated with radiofrequency ablation and transarterial chemoembolization. After these treatments there was tumor progression, with new hyperperfused lesions without evidence of extrahepatic tumor involvement. Our patient refused sorafenib therapy. Therefore, he received twice daily intramuscular injections of Auron Misheil Therapy on an outpatient basis for two months. Partial remission of the hepatic lesions was observed eight weeks after the start of treatment, and confirmed four weeks later. Unfortunately, at that time our patient refused therapy due to dizziness. During follow-up two target lesions remained stable, but one lesion increased in size. At the latest follow-up, one year later, there was still tumor control.

**Conclusion:**

While the mechanisms underlying the antitumor effects of Auron Misheil Therapy are not fully understood, stable disease and remissions have been observed in different types of tumors, including hepatocellular carcinoma.

## Introduction

Hepatocellular carcinoma (HCC) is currently the fifth most common tumor, with 500,000 to one million new cases worldwide per year and 600,000 deaths [1]. In Western countries over 80% of HCCs arise in a cirrhotic liver. Cirrhosis in a setting of chronic liver cell injury, with inflammation, hepatocyte necrosis and regeneration, is a major risk for hepatocyte dedifferentiation and HCC development [2,3]. HCCs are often asymptomatic during early stages. Therefore, the majority of patients (over 80%) show advanced disease or unresectable tumors. Even after successful resection, the recurrence can be as high as 50% at two years [4]. HCCs are mostly resistant to chemotherapy [5,6] and express the multidrug-resistant gene *MDR-1 *[7]. Unfortunately, effective treatment options for advanced HCC are still limited, despite numerous clinical studies with most chemotherapeutic agents. Response rates are low and the response duration is typically short [8,9]. Currently, sorafenib is the only licensed therapy for the treatment of advanced HCC [10]. Recent advances in the understanding of molecular hepatocarcinogenesis include key carcinogenic pathways, such as increased angiogenesis, aberrant signal transduction and dysregulated cell cycle control [11-14].

HCC often develops in association with chronic liver inflammation caused by different risk factors, such as either hepatitis B or C virus infection, alcohol-induced liver injury or obesity-induced fat accumulation. Associated with most HCC risk factors is an increased circulating interleukin-6 (IL-6) level that functions, amongst other factors, as the best predictor of rapid progression from viral hepatitis to HCC in humans [15].

Since proliferation and angiogenesis resemble wound healing and inflammation, anti-inflammatory agents have been evaluated as antitumor agents. In this context, Auron Misheil Therapy (AMT) was developed to add to the existing cancer treatment options. AMT is a fixed combination of aqueous chamomile extract supplemented with calcium, vitamins, the antihistamine chlorpheniramine and human insulin. Gender (men have a higher risk than women), obesity and diabetes are HCC risk factors, and this seems to be due to alterations in the metabolism of endogenous hormones, including sex steroids, insulin and the insulin-like growth factor (IGF) and IGF receptor (IGFR) system [16]. AMT was tested against 56 human tumor cell lines *in vitro*, in a clonogenic assay in 98 patient-derived xenografts, and in *in vivo *studies [17]. *In vitro *cytotoxic activity was highest in cervical cancer and colon cancer as well as in glioblastomas. *In vivo*, AMT showed some antitumor activity in tumor xenograft models of colon and mammary cancer, and in immune stimulation via induction of IL-6 and tumor necrosis factor (TNF)-alpha in human peripheral blood mononuclear cell (PBMCs) [18]. Clinical pilot studies have been initiated in women with advanced cervical cancer and in patients with various solid tumors. Since data from large clinical trials are not yet available, case reports may provide evidence for successful use of AMT.

## Case Presentation

A 78-year-old male Caucasian patient presented at our clinic with multifocal HCC in a cirrhotic liver, Child A due to HCV genotype I infection. Prior to AMT treatment our patient was treated with radiofrequency ablation (RFA) and four transarterial chemoembolizations (TACE) over a four-year period. Due to tumor progression, further TACE or local ablation therapies were no longer viable.

At that time our patient refused sorafenib therapy due to the well documented side effects, such as fatigue, diarrhea, hand-foot-syndrome and others. Therefore, he was included in an ongoing AMT trial. Physical examination revealed an enlarged liver, no clinical signs of ascites or hepatic encephalopathy, no peripheral edema but a slight scleral icterus. Laboratory tests showed a normal blood count, an international normalized ratio of 0.95, albumin 3.5g/dL, bilirubin 1.9 mg/dL, aspartate transaminase 148U/L and alanine transaminase 96U/L. His α-fetoprotein level was elevated (29.5ng/mL) and remained so throughout follow-up.

Twice daily intramuscular injections of AMT were started on an outpatient basis for two months.

**Figure 1:**
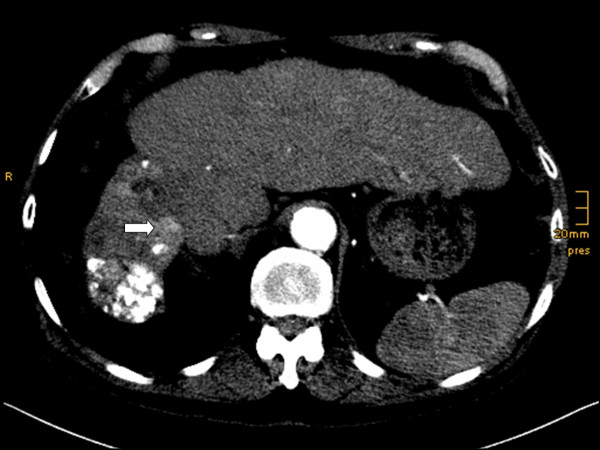
**Baseline CT abdomen scan**. Early arterial examination time, showing liver cirrhosis after transarterial chemoembolization with hypotrophy of his right liver lobe and hypertrophy of his left liver lobe. Lipiodol remnants in the right lobe. Arrow: TL1 in segment 8, size 20 mm.

A baseline computed tomography (CT) scan of his abdomen performed prior to the initiation of AMT identified three target lesions (TL) that fulfilled the Response Evaluation Criteria In Solid Tumors (RECIST) criteria. Two lesions had increased in size since the previous CT scan of his abdomen, performed two months before at the end of the last treatment (Figure 1). TL1 was situated in the right dorsal liver margin, segment 8, and measured 20 mm (previously 12 mm). TL2 was in the right lateral margin of segment 4b, and measured 12 mm (no change from previous CT). TL3 was in the anterior margin of segment 4b, and measured 12 mm (previous 6 mm).

The right liver lobe showed marked atrophy, whereas the left lobe revealed hypertrophy. No extrahepatic manifestations were discovered. His peri-aortacaval lymph nodes were slightly enlarged.

**Figure 2:**
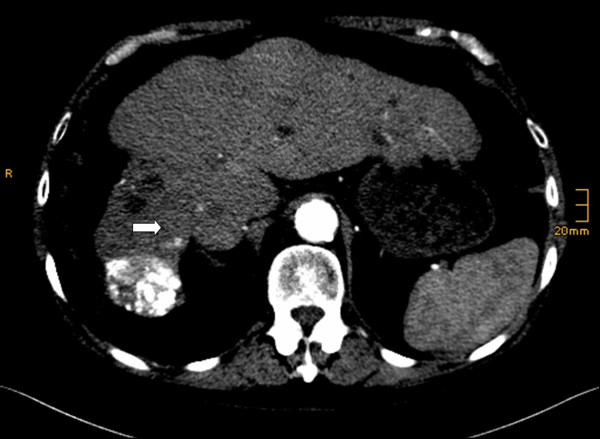
**Control CT abdomen scan after eight weeks of AMT treatment**. Early arterial examination time, showing liver cirrhosis after transarterial chemoembolization. TL1 disappeared.

After eight weeks of AMT treatment the first tumor staging was performed and his abdomen and chest CT scans showed a partial remission. Both target lesions T1 and T2 had disappeared. TL3 had shrunk to 7 mm (40% reduction) with a contrast medium enhancement in the boundary area of the lesion. Again, no extrahepatic involvement was discovered (Figure 2).

According to RECIST, remission has to be confirmed by a second scan at least four weeks later. This scan confirmed the partial remission. The HCC volume in his liver had further decreased with unchanged morphology, in other words, atrophy of the right lobe and hypertrophy of the left lobe. TL1 and TL2 were still invisible, while TL 3 was unchanged in size. In his chest CT scan there was no evidence of lung metastases. Neither bone involvement nor any other extrahepatic manifestation was discovered. Our patient complained about dizziness during AMT and requested to terminate treatment.

Six weeks later, a CT scan revealed several new early arterial hyperperfusion areas in both lobes of his liver, for example, a lesion with 24 mm diameter was found at the right dorsal margin of segment 8. The two defined lesions TL1 and TL2 that had been in complete remission remained in remission. TL3 that had been in partial remission had increased in size to 16 mm. Adrenal involvement was suspected, but lung, spleen, kidney, bones and lymph nodes were free of metastases. Unfortunately, our patient refused to restart AMT despite the remarkable previous tumor control.

Three months later, a follow-up CT scan showed continued tumor control. TL3 had again disappeared, despite having shrunk and re-grown in the preceding months. The other lesions TL1 and TL2 remained in remission. The adrenal metastasis was unchanged and no further tumor progress was observed.

**Figure 3:**
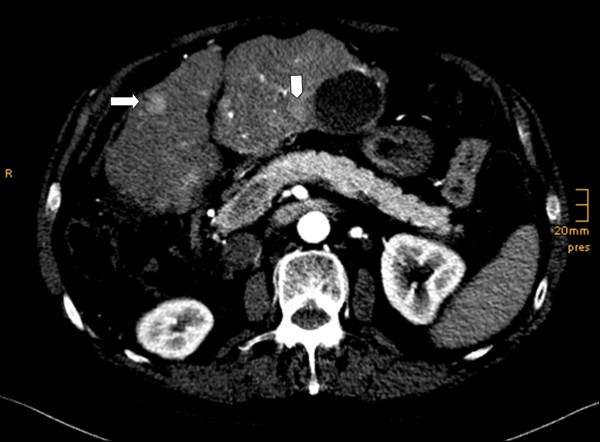
**Follow up CT scan six months later**. Early arterial examination time scan revealing recurrence of TL3, size 11 mm (arrow) and a new lesion next to a cyst of size 12 mm in liver segment 4b (arrow head).

Again, three months after that, progressive disease was once again documented by CT with recurrence of TL3 with a size of 11 mm and several new lesions in both liver lobes up to 22 mm (Figure 3). There were no new extrahepatic metastases except for the adrenal lesion that was unchanged in size. Our patient now agreed to sorafenib therapy.

## Discussion

This case demonstrates that the HCC regression in this patient was closely related to AMT. At week eight of AMT, HCC remission was evident and continued for some time after the treatment was stopped. Not surprisingly, HCC recurred after withdrawal of the treatment. While the molecular basis of the efficacy of AMT is not known in detail, inflammation and immune activation modulated by stress-responsive pathways may be involved [18]. Diverse cellular functions, ranging from differentiation and proliferation to migration and inflammation, are regulated by mitogen-activated protein kinase (MAPK) signaling. The pathway modulates numerous cellular responses through a wide range of activating factors [19]. Pro-tumoral inflammation and the role of intrinsic, oncogene-driven pathways have also been described [20]. In addition, inflammatory processes as well as the epithelial-mesenchymal transitions occur in HCC cells to facilitate their dissemination and are related to cell survival [21].

A central event for the induction of chronic liver disease and the promotion of liver fibrosis, and likely for liver cancer, is inflammation [22]. It has been shown that at least 20% of all cancers developed as a consequence of infection and chronic inflammation. But even cancers not induced in association with chronic inflammation show extensive inflammatory infiltrates, with high cytokine expression levels in the tumor microenvironment [23]. Amongst these cytokines are growth and survival factors that act on premalignant cells [24], stimulate angiogenesis, tumor progression and metastasis, and also maintain tumor-promoting inflammation [25-27]. Pro-inflammatory cytokines, such as TNF and IL-6, can influence all stages of tumor development, including initiation, promotion, progression and metastasis [28,29]. In more than 50% of all cancers, an aberrant activation of nuclear factor kappa-light-chain-enhancer of activated B cells and/or signal transducer and activator of transcription 3 is found, which makes premalignant and fully transformed cells resistant to apoptosis and induces their rate of proliferation, thereby increasing tumor growth [27,30].

Furthermore, necrotic cells, which release their contents-- including pro-inflammatory signals-- into the surrounding tissue microenvironment, can recruit inflammatory cells of the immune system, which are capable of surveying the extent of tissue damage and removing associated necrotic debris [23]. There is evidence that these immune inflammatory cells can be actively tumor-promoting by fostering angiogenesis, cancer cell proliferation and invasiveness [23].

Anti-inflammatory activities, especially those resulting from natural compounds rather than chemicals, may interfere with one or several pathways. AMT contains both natural (chamomile extract) and chemically defined (chlorpheniramine) anti-inflammatory compounds.

The major antiproliferative effect of AMT is mediated by the chlorpheniramine component. Among other mechanisms, chlorpheniramine and structurally related compounds control proliferation by binding to the translationally controlled tumor protein [31]. Like AMT, octreotide functions through multiple mechanisms and receptors inhibiting several secretory and proliferative responses. Within this process, G-protein coupled receptors play a central role by disrupting diverse signal transduction pathways like inhibition of adenylate and guanylate cyclase, modulation of ionic conductance channels and protein dephosphorylation [32]. Somatostatin and its analogues inhibit not only the proliferation of normal and neoplastic cells [33,34], including the hepatocellular cell line Hep G2 [35], but also a variety of experimental tumors. This is mediated by the inhibition of growth arrest through the modulation of MAPK and the induction of G1 cell cycle arrest, as well as by an apoptotic effect through activation of p53 and Bax protein. These actions are mediated directly by somatostatin receptors present on tumor cells and indirectly via somatostatin receptors located on non-tumor cell targets [36,37].

Furthermore, several lines of evidence indicate a role of insulin, IGF and its receptor IGFR in hepatocarcinogenesis [13,14,16,17]. In particular, the Ras/Raf-protein family seems to play a major role in HCC development. This intracellular signaling pathway involves ligands binding to tyrosine kinase receptors, such as the epidermal growth factor receptor and the IGFR. This activates Ras, which in turn activates serine threonine kinases of the Raf-family [38]. Pre-clinical studies of AMT demonstrated cytotoxic activity in a variety of tumor types. As discussed, AMT has shown *in vivo *some antitumor activity in tumor xenograft models of breast and colon cancer as well as immune stimulatory effects in human PBMCs [17]. Clinical studies of AMT are only at their beginning, but tend to confirm the preclinical data. In a pilot study in women with advanced cervical cancer, AMT was well tolerated and improved the quality of life [39]. Interestingly, the presented patient complained about dizziness during AMT treatment. The overall adverse events (AE) of AMT were investigated in an interim analysis, in which no relevant differences were observed between the AMT groups and the placebo group. Under treatment with AMT, approximately one-quarter of the patients experienced AEs (single-blinded AMT: five out of 21 patients (23.8%) experienced 14 events; open AMT: four out of 14 patients (28.6%) experienced five events), involving hypoglycemia, eosinophilia, injection site pain, sciatica and rash. The majority of these events (11 of the 19 events classified as being possibly related to AMT) were rated as mild to moderate. The remaining events were classified as severe (seven events, all hypoglycemia) or life-threatening (one event: hypoglycaemia; also to be classified as serious). Two of these 19 events were classified as 'serious' (both hypoglycemia). The outcome of all 19 events was 'recovered without sequelae'. With the exception of eosinophilia (one mild event) the other AEs described above as being possibly related to the study drug are largely consistent with the known safety profile of AMT.

In patients with stage IIIb or IVa cervical cancer eight out of fifteen (53%) were clinical responders at week 12. One patient had a partial response and 11 patients had stable disease based on RECIST criteria.

Our patient participated in a phase II study of AMT in patients with various solid tumors. Pivotal clinical data on the efficacy of AMT thus tend to support the concept of pleiotropic effects that might also explain the efficacy in different types of tumors.

## Conclusion

Within this case report a stable disease and remissions have been observed in HCC, although the mechanisms of the antitumor effects of AMT are not fully understood. Patients with advanced HCC need not only an effective therapy, but one which does not affect liver function given the preexisting cirrhosis. In this context, AMT might be a therapeutic alternative, with little toxicity compared to cytostatic drugs, provided that its efficacy is confirmed in randomized clinical HCC trials.

However, more results from controlled clinical trials with AMT are needed in order to substantiate this possible treatment alternative.

## Abbreviations

AE: adverse event; AMT: Auron Misheil Therapy; CT: computed tomography; HCC: hepatocellular carcinoma; IGF: insulin-like growth factor; IGFR: insulin-like growth factor receptor; IL-6: interleukin-6; MAPK: mitogen-activated protein kinase; PBMC: peripheral blood mononuclear cell; RECIST: Response Evaluation Criteria In Solid Tumors; TACE: transarterial chemoembolization; TL: target lesion; TNF: tumor necrosis factor.

## Consent

Our patient died in the interim, and written informed consent was obtained from the patient's wife for publication of this case report and accompanying images. A copy of the written consent is available for review by the Editor-in-Chief of this journal.

## Competing interests

The authors declare that they have no competing interests.

## Authors' contributions

JSS wrote the manuscript. JH performed the clinical care and was the treating physician. ZS did the radiology analyses. DR and AD wrote the manuscript. HCS performed the clinical care and was the treating physician. HEB is head of the department and designed the study. All authors read and approved the final manuscript.
